# Supplementary material for: Quantitative proteomics analysis reveals possible anticancer mechanisms of 5’-deoxy-5’-methylthioadenosine in cholangiocarcinoma cells
Source: PLoS One. 2024 Jun 26;19(6):e0306060. doi: 10.1371/journal.pone.0306060 (PMC11206958; doi:10.1371/journal.pone.0306060)
Supplement: S1 Table — (DOCX) [file pone.0306060.s001.docx]

| **S1 Table. Differentially expressed proteins in KKU-213A after MTA treatment** | | | | | | | | | |
| --- | --- | --- | --- | --- | --- | --- | --- | --- | --- |
| **UniProt Number** | **Protein names** | **Gene names** | **Expressed value (log2)** | | | | | |  |
|  |  |  | **Control** | | | **MTA treatment** | | |  |
|  |  |  | **C-1** | **C-2** | **C-3** | **M-1** | **M-2** | **M-3** |  |
| A0A669KB37 | Transmembrane protein 253 | TMEM253 | 20.11 | 18.92 | 19.37 | 0.00 | 0.00 | 0.00 |  |
| A6NHJ4 | Zinc finger protein 860 | ZNF860 | 19.65 | 19.91 | 17.68 | 0.00 | 0.00 | 0.00 |  |
| E5RFQ1 | Transcription factor COE1 | EBF1 | 17.46 | 16.94 | 18.19 | 0.00 | 0.00 | 0.00 |  |
| H0YGB8 | Kinesin light chain 1 | KLC1 | 17.63 | 16.88 | 17.73 | 0.00 | 0.00 | 0.00 |  |
| Q8IYS0 | Protein Aster-C | GRAMD1C | 15.90 | 18.26 | 16.96 | 0.00 | 0.00 | 0.00 |  |
| A0A286YFD1 | Fer-1-like protein 5 | FER1L5 | 17.54 | 12.45 | 18.81 | 0.00 | 0.00 | 0.00 |  |
| Q6ZN01 | MEF2-activating motif and SAP domain-containing transcriptional regulator | MAMSTR | 13.19 | 18.03 | 15.48 | 0.00 | 0.00 | 0.00 |  |
| Q9C0D5 | Protein TANC1 (Tetratricopeptide repeat, ankyrin repeat and coiled-coil domain-containing protein 1) | TANC1 | 10.82 | 15.56 | 14.75 | 0.00 | 0.00 | 0.00 |  |
| Q8IZ40 | REST corepressor 2 | RCOR2 | 17.55 | 9.41 | 13.72 | 0.00 | 0.00 | 0.00 |  |
| E5RJP6 | Tetratricopeptide repeat protein 13 | TTC13 | 19.69 | 21.25 | 21.25 | 16.83 | 18.15 | 16.50 |  |
| Q8N1Q1 | Carbonic anhydrase 13 | CA13 | 18.28 | 19.45 | 18.30 | 14.76 | 15.75 | 15.99 |  |
| Q4G0L6 | ZNF668 protein | ZNF668 | 19.59 | 19.31 | 19.93 | 16.37 | 17.07 | 16.27 |  |
| L8E8N7 | Alternative protein PLEKHA2 | PLEKHA2 | 18.19 | 19.31 | 18.43 | 16.29 | 16.52 | 14.89 |  |
| Q5VWT5 | FYN-binding protein 2 | FYB2 | 17.38 | 17.01 | 18.25 | 13.88 | 14.61 | 16.31 |  |
| X6RC68 | Protein zer-1 homolog | ZER1 | 17.89 | 19.30 | 18.77 | 15.98 | 15.84 | 16.34 |  |
| A0A1B0GX35 | Solute carrier organic anion transporter family member 2B1 | SLCO2B1 | 17.67 | 17.69 | 17.74 | 15.23 | 16.57 | 13.58 |  |
| H7C2F4 | Xyloside xylosyltransferase 1 | XXYLT1 | 19.07 | 20.85 | 20.55 | 17.86 | 17.38 | 17.61 |  |
| A0A669KBF2 | Carbamoyl-phosphate synthase | CPS1 | 20.17 | 21.02 | 19.86 | 16.98 | 19.22 | 17.79 |  |
| Q9Y3F6 | EST00098 protein | EST00098 | 20.41 | 19.63 | 20.69 | 17.51 | 18.11 | 18.16 |  |

| **S1 Table. Differentially expressed proteins in KKU-213A after MTA treatment (continued)** | | | | | | | | |
| --- | --- | --- | --- | --- | --- | --- | --- | --- |
| **UniProt Number** | **Protein names** | **Gene names** | **Expressed value (log2)** | | | | | |
|  |  |  | **Control** | | | **MTA treatment** | | |
|  |  |  | **C-1** | **C-2** | **C-3** | **M-1** | **M-2** | **M-3** |
| Q5T8R2 | Glutathione S-transferase | GSTM5 | 20.43 | 20.00 | 20.20 | 17.64 | 17.82 | 18.47 |
| Q4VXS0 | Chromosome 20 open reading frame 173 | C20orf173 | 18.32 | 19.36 | 19.71 | 17.09 | 17.23 | 16.48 |
| A0A023QFT1 | NADH-ubiquinone oxidoreductase chain 1 | ND1 | 19.97 | 20.38 | 19.82 | 17.92 | 18.05 | 17.65 |
| J9ZVQ3 | Apolipoprotein E | APOE | 19.97 | 21.16 | 21.56 | 17.83 | 19.01 | 19.38 |
| E9PAL5 | Collagen alpha-5(VI) chain | COL6A5 | 20.10 | 19.94 | 19.80 | 18.23 | 17.77 | 17.39 |
| Q8IVE3 | Pleckstrin homology domain-containing family H member 2 | PLEKHH2 | 18.96 | 18.38 | 18.89 | 16.87 | 16.15 | 16.74 |
| A0A1B0GUK1 | Potassium voltage-gated channel subfamily KQT member 2 | KCNQ2 | 18.99 | 18.25 | 18.96 | 16.01 | 16.85 | 16.99 |
| A0A024R6I9 | Serpin peptidase inhibitor, clade A | SERPINA4 | 19.84 | 19.61 | 18.99 | 16.66 | 17.28 | 18.16 |
| B7ZKS5 | TRPM5 protein | TRPM5 | 19.54 | 18.97 | 18.24 | 16.09 | 17.23 | 17.18 |
| A0A1B0GU82 | Gamma-aminobutyric acid receptor subunit alpha-1 | GABRA1 | 21.84 | 20.12 | 20.69 | 19.21 | 19.14 | 18.16 |
| L8E7X8 | Alternative protein CD84 | CD84 | 18.92 | 19.79 | 20.46 | 17.77 | 18.40 | 16.87 |
| Q99575 | Ribonucleases P/MRP protein subunit POP1 | POP1 | 18.77 | 18.22 | 20.25 | 17.68 | 17.14 | 16.31 |
| A0A3B3IS64 | WD repeat-containing protein 27 | WDR27 | 17.63 | 18.05 | 17.40 | 15.29 | 15.77 | 15.90 |
| Q9BZK8 | Ovarian cancer-related protein 1 | OCR1 | 17.74 | 17.91 | 18.96 | 16.40 | 16.37 | 15.74 |
| Q00534 | Cyclin-dependent kinase 6 | CDK6 | 17.88 | 18.32 | 18.99 | 15.72 | 16.86 | 16.52 |
| A7J1Q6 | Variant ATP-binding cassette sub-family B member 4 | ABCB4 | 19.58 | 18.99 | 19.15 | 17.27 | 17.93 | 16.50 |
| A0A3B3ISE1 | Uncharacterized protein C9orf153 | - | 19.56 | 19.83 | 20.94 | 18.84 | 18.48 | 17.00 |
| Q6AW98 | Uncharacterized protein DKFZp686P13170 | - | 20.99 | 21.20 | 20.68 | 19.20 | 19.72 | 17.95 |
| F6XZD3 | Ankyrin repeat domain-containing protein 35 | ANKRD35 | 17.00 | 17.02 | 16.65 | 15.65 | 14.53 | 14.50 |
| A0N4W2 | Possible J 50 gene segment | Tcr-alpha | 20.30 | 19.29 | 19.83 | 17.61 | 18.18 | 17.67 |
| Q8N7B9 | EF-hand calcium-binding domain-containing protein 3 | EFCAB3 | 20.27 | 21.95 | 21.13 | 19.69 | 18.58 | 19.14 |
| A0A0B4J1V9 | Helicase, lymphoid-specific, isoform CRA_b | HELLS | 20.11 | 19.97 | 21.17 | 19.46 | 17.96 | 17.96 |
| Q5T0V4 | Slit homolog 1 protein | SLIT1 | 19.83 | 19.66 | 20.24 | 18.09 | 17.22 | 18.55 |
| B2RBU0 | PRP18 homolog (Pre-mRNA-splicing factor 18) | PRPF18 | 19.78 | 20.37 | 21.00 | 18.23 | 18.69 | 18.40 |
| H0YC49 | Histidine triad nucleotide-binding protein 1 | HINT1 | 19.11 | 18.51 | 19.57 | 17.60 | 17.00 | 16.78 |
| B8ZZK0 | Rho GTPase-activating protein 15 | ARHGAP15 | 20.59 | 19.77 | 19.52 | 17.33 | 19.10 | 17.65 |
| Q96Q36 | ALS2CR11 protein | ALS2CR11 | 19.79 | 20.19 | 19.34 | 16.80 | 17.84 | 18.91 |
| A0A1C7CYW9 | Protein monoglycylase TTLL8 | TTLL8 | 19.60 | 21.07 | 19.96 | 18.00 | 19.00 | 17.88 |
| A0A1B0GUE5 | Voltage-dependent P/Q-type calcium channel subunit alpha-1A | CACNA1A | 18.19 | 18.99 | 18.32 | 16.54 | 16.92 | 16.31 |
| H0YBC5 | Thyroglobulin | TG | 21.39 | 21.80 | 20.95 | 19.15 | 19.79 | 19.56 |
| D3DTQ7 | HCG2036579, isoform CRA_b | hCG_2036579 | 17.86 | 18.27 | 17.58 | 14.97 | 16.64 | 16.56 |
| Q5SRI9 | Glycoprotein endo-alpha-1,2-mannosidase | MANEA | 17.06 | 16.67 | 17.29 | 14.75 | 15.60 | 15.14 |
| A0A087WWM0 | Trafficking protein particle complex subunit | TRAPPC3 | 20.23 | 20.42 | 18.90 | 18.28 | 17.51 | 18.26 |
| Q8TDC0 | Myozenin-3 | MYOZ3 | 19.11 | 20.83 | 20.17 | 17.78 | 18.41 | 18.42 |
| Q24JQ3 | E2F3 protein | E2F3 | 18.36 | 19.58 | 19.13 | 16.55 | 16.78 | 18.27 |
| A0A024R914 | Centrosomal protein 350kDa, isoform CRA_a | CEP350 | 19.90 | 20.76 | 21.01 | 17.81 | 19.04 | 19.35 |
| A0A590UK21 | Troponin T, cardiac muscle | TNNT2 | 17.00 | 17.40 | 17.31 | 16.02 | 15.98 | 14.24 |
| Q9P287 | BRCA2 and CDKN1A-interacting protein | BCCIP | 20.92 | 21.81 | 21.29 | 19.40 | 19.76 | 19.39 |
| A0A494C0R4 | Serine/threonine-protein kinase TBK1 | TBK1 | 20.91 | 20.38 | 20.65 | 18.12 | 20.07 | 18.31 |
| H7C5D8 | Zinc finger protein ZIC 1 | ZIC1 | 21.95 | 22.32 | 21.92 | 20.31 | 20.50 | 19.99 |
| A0A0D9SFZ2 | Cyclin-dependent kinase 8 | CDK8 | 20.66 | 20.87 | 21.38 | 18.84 | 18.96 | 19.75 |
| B2R7Q9 | cDNA, FLJ93562 | - | 21.21 | 19.84 | 19.82 | 17.66 | 19.05 | 18.80 |
| E5KTI5 | Endonuclease III-like protein 1 | NTHL1 | 21.12 | 20.38 | 19.46 | 18.31 | 18.72 | 18.67 |
| A0A7P0T922 | Tetratricopeptide repeat protein 39A | TTC39A | 19.62 | 18.71 | 18.52 | 16.24 | 17.40 | 17.98 |
| K7ELB4 | PRELI domain-containing protein 3A | PRELID3A | 18.30 | 18.00 | 18.30 | 15.43 | 16.75 | 17.29 |
| Q86WI6 | Integrase interactor 1 protein isoform F | SMARCB1 | 21.04 | 22.28 | 20.77 | 19.12 | 19.65 | 20.23 |
| A0A7P0T8V7 | Sushi domain-containing protein 4 | SUSD4 | 20.54 | 21.30 | 20.05 | 18.44 | 18.68 | 19.70 |
| Q6NZY7 | Cdc42 effector protein 5 | CDC42EP5 | 18.51 | 19.19 | 19.15 | 17.01 | 17.70 | 17.10 |
| B7ZMG3 | KIAA1529 protein | KIAA1529 | 18.97 | 19.41 | 18.87 | 16.85 | 17.84 | 17.53 |
| A0A075XDP4 | NADH-ubiquinone oxidoreductase chain 3 | ND3 | 19.95 | 19.98 | 19.39 | 17.28 | 18.80 | 18.22 |
| A6NNM3 | RIMS-binding protein 3B | RIMBP3B | 17.37 | 16.89 | 17.70 | 16.19 | 14.56 | 16.22 |
| A0A087X0H5 | Growth hormone receptor | GHR | 17.81 | 17.83 | 18.43 | 16.36 | 15.47 | 17.32 |
| E5KRP6 | Spastin | SPAST | 20.33 | 18.87 | 20.36 | 17.97 | 18.83 | 17.85 |
| Q9BX84 | Transient receptor potential cation channel subfamily M member 6 | TRPM6 | 18.74 | 19.44 | 18.22 | 17.39 | 16.95 | 17.14 |
| K7EMI9 | Coiled-coil domain-containing protein 106 | CCDC106 | 17.69 | 18.41 | 18.28 | 16.94 | 16.75 | 15.79 |
| Q6ZMH1 | cDNA FLJ23933 fis, clone COL07392 | - | 20.29 | 21.17 | 20.37 | 18.76 | 19.44 | 18.74 |
| Q8IX90 | Spindle and kinetochore-associated protein 3 | SKA3 | 19.55 | 19.95 | 20.32 | 17.65 | 18.46 | 18.87 |
| R9UH47 | Collagen type VIII alpha 2 | COL8A2 | 21.02 | 20.00 | 19.64 | 19.14 | 17.88 | 18.80 |
| Q53TK5 | Uncharacterized protein ADAM23 | ADAM23 | 16.88 | 17.24 | 15.93 | 14.35 | 15.44 | 15.45 |
| Q9BYH1 | Seizure 6-like protein | SEZ6L | 19.30 | 18.08 | 19.35 | 17.31 | 16.80 | 17.82 |
| Q8NCA9 | Zinc finger protein 784 | ZNF784 | 19.74 | 19.85 | 19.09 | 17.72 | 18.49 | 17.68 |
| A0A2R8Y4Z8 | Coiled-coil domain-containing protein 9 | CCDC9 | 17.40 | 18.18 | 18.56 | 16.14 | 16.15 | 17.06 |
| A0A024RDK4 | Integrator complex subunit 12 | INTS12 | 19.81 | 19.89 | 19.09 | 17.96 | 17.80 | 18.26 |
| Q99952 | Tyrosine-protein phosphatase non-receptor type 18 | PTPN18 | 19.72 | 17.96 | 18.49 | 17.44 | 16.97 | 17.00 |
| A0A087WSZ0 | Immunoglobulin kappa variable 1D-8 | IGKV1D-8 | 17.55 | 18.08 | 18.63 | 16.16 | 16.66 | 16.70 |
| Q7Z641 | HMGXB4 protein | HMGXB4 | 18.50 | 19.02 | 18.20 | 16.28 | 17.27 | 17.43 |
| A6MW40 | PKDREJ | PKDREJ | 20.60 | 19.80 | 20.67 | 19.17 | 18.61 | 18.60 |
| F8W9E7 | Engulfment and cell motility protein 3 | ELMO3 | 19.72 | 20.80 | 19.01 | 18.01 | 18.67 | 18.18 |
| H3BPP9 | BAI1-associated protein 3 | BAIAP3 | 18.13 | 17.77 | 17.93 | 16.19 | 16.83 | 16.12 |
| Q15643 | Thyroid receptor-interacting protein 11 | TRIP11 | 17.29 | 17.86 | 17.95 | 16.35 | 16.67 | 15.43 |
| B7Z8V5 | cDNA FLJ51186, highly similar to Homo sapiens ADP-ribosylation factor-like 6 interacting protein 2 (ARL6IP2), mRNA | - | 17.82 | 17.30 | 17.44 | 16.56 | 15.01 | 16.34 |
| A0A5C2GET4 | IGH + IGL c389_heavy_IGHV4-39_IGHD1-26_IGHJ6 (Fragment) | - | 21.46 | 20.97 | 21.00 | 19.81 | 19.05 | 19.94 |
| H0Y7V4 | Dynein heavy chain 8, axonemal | DNAH8 | 20.97 | 20.90 | 20.72 | 19.41 | 19.43 | 19.13 |
| Q05CL3 | BCAS1 protein | BCAS1 | 18.42 | 18.37 | 18.82 | 16.71 | 17.65 | 16.65 |
| F5H1A5 | Splicing factor, suppressor of white-apricot homolog | SFSWAP | 20.72 | 21.22 | 21.05 | 18.77 | 19.59 | 20.04 |
| B4F4S5 | Rhesus blood group, D antigen | RHD | 20.27 | 19.94 | 20.30 | 18.07 | 19.13 | 18.74 |
| A8K4A5 | cDNA FLJ77482, highly similar to Human atrial natriuretic peptide clearance receptor (ANP C-receptor) mRNA | - | 20.88 | 21.59 | 20.33 | 19.18 | 19.78 | 19.29 |
| C9JRW1 | Suppressor of tumorigenicity 7 protein | ST7 | 19.32 | 19.42 | 19.55 | 17.50 | 18.92 | 17.34 |
| A0A024R179 | Nuclear cap binding protein subunit 1, 80kDa, isoform CRA_a | NCBP1 | 19.69 | 20.14 | 19.94 | 17.92 | 18.00 | 19.33 |
| P35716 | Transcription factor SOX-11 | SOX11 | 19.19 | 18.96 | 18.94 | 16.92 | 18.31 | 17.33 |
| Q8IZQ8 | Myocardin | MYOCD | 18.30 | 17.91 | 17.67 | 16.09 | 17.04 | 16.25 |
| Q9NW25 | Single stranded DNA binding protein 3, isoform CRA_c | SSBP3 | 19.34 | 20.37 | 20.34 | 18.13 | 19.14 | 18.31 |
| A0A7S5C2L1 | IGH c2737_heavy_IGHV1-69_IGHD6-13_IGHJ4 | - | 17.49 | 17.43 | 17.16 | 15.99 | 16.08 | 15.56 |
| Q9Y4S0 | Uncharacterized protein DKFZp570I0164 | - | 18.51 | 18.29 | 18.32 | 16.93 | 17.39 | 16.37 |
| A0A2R8Y4H4 | Leucine-rich repeat-containing protein 9 | LRRC9 | 17.52 | 17.26 | 17.96 | 16.48 | 15.73 | 16.14 |
| B4E071 | cDNA FLJ54215 | - | 18.97 | 19.10 | 17.82 | 17.40 | 17.15 | 16.97 |
| Q5TC63 | Growth hormone-regulated TBC protein 1 | GRTP1 | 17.88 | 18.37 | 18.21 | 16.46 | 16.71 | 16.91 |
| Q8TD08 | Mitogen-activated protein kinase 15 | MAPK15 | 19.90 | 20.33 | 20.18 | 18.36 | 19.24 | 18.47 |
| Q53YE2 | Syntaxin 3A | STX3A | 19.88 | 20.14 | 19.17 | 17.53 | 18.48 | 18.84 |
| Q7Z4C9 | MSTP140 | - | 18.42 | 17.95 | 17.68 | 15.95 | 16.82 | 16.94 |
| A0A0J9YXV8 | Kell blood group glycoprotein | KEL | 19.09 | 18.61 | 19.33 | 17.16 | 17.24 | 18.31 |
| Q8N4N3 | Kelch-like protein 36 | KLHL36 | 18.89 | 19.04 | 18.07 | 17.75 | 17.19 | 16.76 |
| A0A6B7G0S0 | MHC class II antigen | HLA-DQB1 | 19.46 | 18.56 | 19.31 | 16.98 | 17.82 | 18.25 |
| G5E9W0 | Phospholipase A1 member A | PLA1A | 20.14 | 19.39 | 20.73 | 18.70 | 18.14 | 19.16 |
| B2R8Y4 | cDNA, FLJ94117, highly similar to Homo sapiens actinin, alpha 3 (ACTN3), mRNA | - | 18.03 | 17.15 | 17.23 | 15.86 | 16.57 | 15.74 |
| A6N6J7 | [Histone H3]-trimethyl-L-lysine(4) demethylase | JARID1C | 21.10 | 21.48 | 20.93 | 19.47 | 20.29 | 19.53 |
| P20718 | Granzyme H | GZMH | 18.57 | 18.99 | 18.96 | 16.99 | 17.66 | 17.67 |
| B3KSD3 | cDNA FLJ36024 fis, clone TESTI2016667, highly similar to Homo sapiens zinc finger CCCH-type containing 12A (ZC3H12A), mRNA | - | 17.21 | 17.96 | 16.95 | 16.12 | 15.39 | 16.40 |
| V5IRT4 | Mitochondrial nucleoid factor 1 | UQCC2 | 20.82 | 20.59 | 19.48 | 18.84 | 19.25 | 18.61 |
| P54750 | Calcium/calmodulin-dependent 3',5'-cyclic nucleotide phosphodiesterase 1A | PDE1A | 17.64 | 16.98 | 17.74 | 16.76 | 15.83 | 15.59 |
| E7EQZ4 | Survival motor neuron protein | SMN1 | 19.83 | 20.24 | 19.46 | 18.23 | 19.23 | 17.95 |
| H7BY53 | Dynein regulatory complex protein 8 | EFCAB2 | 18.93 | 19.55 | 19.45 | 18.32 | 17.84 | 17.66 |
| B3KUK7 | Multifunctional fusion protein [Includes: Katanin p60 ATPase-containing subunit A1 (Katanin p60 subunit A1) (EC 5.6.1.1) (p60 katanin); Katanin p60 ATPase-containing subunit A-like 1 (Katanin p60 subunit A-like 1) (p60 katanin-like 1)] | KATNA1 | 17.75 | 18.43 | 17.21 | 15.84 | 16.90 | 16.58 |
| D7P9H2 | ATP synthase subunit a | ATP6 | 18.13 | 18.44 | 18.26 | 16.67 | 17.00 | 17.09 |
| H7C4G8 | 5'-nucleotidase domain-containing protein 2 | NT5DC2 | 17.43 | 18.59 | 18.12 | 17.38 | 16.43 | 16.30 |
| Q6ZSA8 | Putative uncharacterized protein FLJ45684 | - | 19.20 | 19.47 | 19.62 | 18.56 | 18.00 | 17.70 |
| I3NI40 | Uncharacterized protein | - | 19.09 | 18.40 | 18.69 | 17.31 | 17.70 | 17.14 |
| Q03164 | Histone-lysine N-methyltransferase 2A | KMT2A | 20.90 | 20.34 | 20.61 | 19.02 | 19.00 | 19.81 |
| A0A1W2PP17 | STE20-related kinase adapter protein alpha | STRADA | 18.22 | 17.16 | 17.50 | 15.96 | 16.97 | 15.94 |
| Q8IUQ0 | Clavesin-1 | CLVS1 | 20.43 | 21.16 | 20.53 | 19.18 | 19.24 | 19.68 |
| H7C2I5 | Polypeptide N-acetylgalactosaminyltransferase 13 | GALNT13 | 18.13 | 17.94 | 18.76 | 17.08 | 16.33 | 17.43 |
| Q96A28 | SLAM family member 9 | SLAMF9 | 19.98 | 20.69 | 19.49 | 18.24 | 19.04 | 18.92 |
| B4DMB6 | cDNA FLJ56698, highly similar to CASP8 and FADD-like apoptosis regulator | - | 20.26 | 21.01 | 21.56 | 19.52 | 19.80 | 19.56 |
| B4E174 | cDNA FLJ55605 | - | 20.96 | 20.39 | 20.43 | 18.45 | 19.47 | 19.91 |
| E9PPE0 | NADH dehydrogenase [ubiquinone] flavoprotein 1, mitochondrial | NDUFV1 | 19.10 | 18.56 | 18.41 | 17.17 | 17.29 | 17.67 |
| H7BZ37 | BCL-6 corepressor | BCOR | 15.60 | 16.29 | 16.37 | 14.74 | 15.18 | 14.41 |
| E5RIF7 | Tubby-related protein 2 | TULP2 | 20.06 | 19.73 | 18.91 | 18.05 | 18.79 | 17.95 |
| A0A024R0N6 | Spectrin beta chain | SPTBN4 | 18.24 | 19.35 | 18.86 | 17.50 | 17.18 | 17.87 |
| A0A024RE06 | DNA ligase | LIG4 h | 20.26 | 20.83 | 20.19 | 18.86 | 18.72 | 19.82 |
| Q8TCC6 | DMGDH protein | DMGDH | 20.80 | 21.03 | 19.84 | 19.02 | 19.55 | 19.23 |
| Q9H0M0 | NEDD4-like E3 ubiquitin-protein ligase WWP1 | WWP1 | 18.66 | 18.98 | 18.58 | 16.99 | 17.49 | 17.88 |
| A0A166HMW0 | NADPH-dependent retinol dehydrogenase/reductase-like protein 2 isoform S4 | DHRS4L2 | 19.90 | 20.55 | 19.56 | 18.55 | 18.60 | 19.01 |
| C6EVS4 | Calpain-3 | CAPN3 | 21.51 | 21.71 | 20.96 | 19.50 | 20.17 | 20.66 |
| Q5SZR1 | 39S ribosomal protein L9, mitochondrial | MRPL9 | 18.16 | 17.70 | 18.48 | 16.18 | 17.27 | 17.04 |
| Q9Y623 | Myosin-4 | MYH4 | 17.37 | 17.87 | 18.08 | 16.17 | 16.88 | 16.42 |
| A1A4H0 | VPS16 protein | VPS16 | 20.01 | 20.15 | 19.65 | 19.03 | 18.53 | 18.41 |
| Q9Y2H9 | Microtubule-associated serine/threonine-protein kinase 1 | MAST1 | 21.09 | 21.58 | 21.12 | 20.30 | 19.87 | 19.79 |
| C9JZC2 | Zinc finger protein 621 | ZNF621 | 19.21 | 18.98 | 19.24 | 17.55 | 18.18 | 17.87 |
| K7ENM7 | Uncharacterized protein | - | 19.59 | 20.14 | 20.17 | 18.55 | 18.92 | 18.65 |
| A0A024R7N5 | Kelch-like 26 (Drosophila), isoform CRA_a | KLHL26 | 20.71 | 21.05 | 21.15 | 19.14 | 19.62 | 20.37 |
| Q86YX8 | Hypothetical drug-resistance-associated protein | - | 18.29 | 18.38 | 18.76 | 17.05 | 17.62 | 17.01 |
| Q9UMQ6 | Calpain-11 | CAPN11 | 18.34 | 17.64 | 17.32 | 15.95 | 16.98 | 16.62 |
| Q9NQZ6 | Zinc finger C4H2 domain-containing protein | ZC4H2 | 19.85 | 20.51 | 20.84 | 19.23 | 19.44 | 18.79 |
| B3KQ62 | cDNA FLJ32946 fis, clone TESTI2007872, weakly similar to INTRACELLULAR PROTEIN TRANSPORT PROTEIN USO1 | - | 20.75 | 20.48 | 20.78 | 18.82 | 19.45 | 20.02 |
| F8WEI0 | Pyrroline-5-carboxylate reductase 3 | PYCR3 | 20.77 | 20.88 | 19.98 | 18.85 | 19.39 | 19.68 |
| Q49A97 | TTC17 protein | TTC17 | 18.05 | 18.84 | 17.97 | 16.59 | 17.49 | 17.09 |
| Q14008 | Cytoskeleton-associated protein 5 | CKAP5 | 21.05 | 20.27 | 21.40 | 19.11 | 19.96 | 19.96 |
| C9J198 | Lebercilin-like protein | LCA5L | 20.09 | 19.91 | 20.54 | 18.83 | 18.64 | 19.37 |
| Q9H788 | SH2 domain-containing protein 4A | SH2D4A | 17.99 | 17.52 | 17.45 | 16.79 | 16.06 | 16.42 |
| Q9NYV4 | Cyclin-dependent kinase 12 | CDK12 | 18.54 | 19.35 | 18.24 | 17.22 | 17.80 | 17.42 |
| Q9NTH0 | HCG1791715 | - | 22.30 | 21.61 | 20.99 | 20.03 | 20.66 | 20.53 |
| Q6ZRT1 | cDNA FLJ46124 fis, clone TESTI2040372 | - | 21.26 | 21.42 | 20.52 | 19.42 | 20.09 | 20.02 |
| Q2YHU8 | Rhesus blood group D antigen | RHD | 20.77 | 21.02 | 20.76 | 19.12 | 20.09 | 19.67 |
| A0A1S6NEW5 | Cytochrome c oxidase subunit 1 | COX1 | 21.15 | 21.09 | 20.83 | 19.91 | 19.66 | 19.83 |
| Q4VAU9 | Serine/threonine-protein kinase receptor | ACVR2B | 19.44 | 19.06 | 19.10 | 17.60 | 17.64 | 18.71 |
| Q8N774 | cDNA FLJ25966 fis, clone TST05207 | - | 20.57 | 20.01 | 19.64 | 19.27 | 18.38 | 18.91 |
| Q5U058 | Axonal membrane protein GAP-43 | GAP43 | 20.07 | 19.40 | 19.80 | 18.41 | 18.44 | 18.79 |
| A0A0E3X973 | NADH-ubiquinone oxidoreductase chain 4 | ND4 | 18.84 | 19.58 | 18.98 | 17.57 | 18.17 | 18.03 |
| O15554 | Intermediate conductance calcium-activated potassium channel protein 4 | KCNN4 | 21.21 | 21.15 | 20.35 | 19.20 | 20.05 | 19.84 |
| A0A1X7SC74 | CBY1-interacting BAR domain-containing protein 2 | CIBAR2 | 20.44 | 20.07 | 19.62 | 18.50 | 19.30 | 18.73 |
| I6L961 | CCNE2 protein | CCNE2 | 17.56 | 17.42 | 18.14 | 16.85 | 16.42 | 16.25 |
| M0R2X8 | Angiopoietin-related protein 4 | ANGPTL4 | 21.01 | 20.65 | 21.17 | 19.21 | 20.24 | 19.78 |
| J3KP11 | Voltage-dependent R-type calcium channel subunit alpha | CACNA1E | 18.62 | 18.61 | 17.89 | 17.20 | 17.13 | 17.20 |
| A0A5C2GSC6 | IG c1574_heavy_IGHV4-34_IGHD2-21_IGHJ2 | - | 21.37 | 22.09 | 21.42 | 20.09 | 20.32 | 20.89 |
| Q8IZP6 | RING finger protein 113B | RNF113B | 19.33 | 19.01 | 18.29 | 17.38 | 17.97 | 17.69 |
| H0YE68 | Histone-lysine N-methyltransferase NSD3 | NSD3 | 20.15 | 19.75 | 19.62 | 19.27 | 18.21 | 18.47 |
| B4DZJ7 | Transcription elongation factor SPT5 | - | 20.92 | 20.31 | 20.34 | 19.86 | 19.51 | 18.63 |
| Q86W77 | ANKS3 protein | ANKS3 | 19.05 | 17.85 | 18.47 | 17.35 | 16.82 | 17.64 |
| B3KV11 | cDNA FLJ16022 fis, highly similar to Leucine-rich repeat transmembrane neuronalprotein 4 | - | 19.34 | 18.97 | 18.56 | 17.52 | 18.00 | 17.80 |
| F8W111 | Carboxypeptidase M | CPM | 18.46 | 18.43 | 18.97 | 17.44 | 17.29 | 17.60 |
| E9PSD9 | Single Ig IL-1-related receptor | SIGIRR | 21.12 | 21.86 | 20.97 | 20.15 | 20.68 | 19.59 |
| A0A7I8V395 | Polyprotein | VACV | 18.35 | 18.00 | 18.01 | 17.15 | 16.47 | 17.23 |
| A0A024R0Q1 | Zinc finger protein 45, isoform CRA_a | ZNF45 | 19.85 | 20.06 | 19.85 | 18.68 | 18.58 | 19.00 |
| B2LYR2 | CD247 transcript variant 2 | CD247 | 20.23 | 20.44 | 19.62 | 18.43 | 19.37 | 19.00 |
| P16471 | Prolactin receptor | PRLR | 18.90 | 18.60 | 18.23 | 16.68 | 17.77 | 17.79 |
| A0A024R9G1 | Sperm associated antigen 1, isoform CRA_d | SPAG1 | 18.12 | 18.07 | 18.09 | 16.25 | 17.20 | 17.38 |
| E7ESW6 | WD repeat-containing protein 87 | WDR87 | 21.19 | 20.25 | 20.17 | 19.01 | 19.65 | 19.52 |
| Q05CP8 | CCDC6 protein | CCDC6 | 18.00 | 18.55 | 18.24 | 16.83 | 17.10 | 17.42 |
| S4R3I3 | Protein phosphatase 1J | PPM1J | 20.92 | 20.76 | 19.67 | 19.27 | 19.40 | 19.26 |
| M0QX37 | Microtubule-associated protein 1S | MAP1S | 20.84 | 21.01 | 19.79 | 19.23 | 19.38 | 19.62 |
| Q9Y6H8 | Gap junction alpha-3 protein | GJA3 | 18.33 | 18.53 | 19.08 | 16.91 | 17.94 | 17.69 |
| Q8N5H7 | SH2 domain-containing protein 3C | SH2D3C | 21.44 | 20.76 | 20.57 | 19.75 | 19.84 | 19.79 |
| S4R3X0 | PC-esterase domain-containing protein 1A | PCED1A | 19.96 | 19.03 | 19.24 | 18.09 | 18.72 | 18.02 |
| A0A5C2GIU4 | IG c1159_heavy_IGHV1-69_IGHD4-17_IGHJ6 | - | 17.57 | 17.85 | 17.57 | 15.80 | 16.65 | 17.13 |
| B4DWV3 | cDNA FLJ54820, highly similar to Ly6/PLAUR domain-containing protein 5 | - | 19.97 | 19.15 | 18.80 | 18.26 | 18.37 | 17.90 |
| A0A024R6I1 | Checkpoint suppressor 1, isoform CRA_a | CHES1 | 18.16 | 18.48 | 18.62 | 16.89 | 17.01 | 17.98 |
| A0A7N4I390 | NTPase KAP family P-loop domain-containing protein 1 | NKPD1 | 20.71 | 20.10 | 20.28 | 18.72 | 19.31 | 19.67 |
| A6NMV7 | Orexin receptor type 1 | HCRTR1 | 18.86 | 18.03 | 17.88 | 16.89 | 17.49 | 17.02 |
| C9JAE6 | Multidrug and toxin extrusion protein 2 | SLC47A2 | 20.99 | 20.89 | 20.50 | 19.02 | 20.05 | 19.94 |
| B3KTY7 | cDNA FLJ38970 fis, clone NT2RI2002391, highly similar to Homo sapiens thrombospondin, type I, domain containing 1 (THSD1), transcript variant 2, mRNA | - | 19.13 | 19.02 | 18.54 | 17.66 | 17.47 | 18.20 |
| Q9H9L3 | Interferon-stimulated 20 kDa exonuclease-like 2 | ISG20L2 | 20.42 | 20.51 | 20.31 | 18.74 | 19.74 | 19.41 |
| B2R988 | RNA helicase | - | 20.48 | 20.68 | 19.87 | 19.16 | 19.44 | 19.10 |
| A7U7M2 | Prion protein 2 | PRND | 17.70 | 18.31 | 17.52 | 16.50 | 16.47 | 17.22 |
| A0A024RBI8 | G protein-coupled receptor kinase interactor 2, isoform CRA_a | GIT2 | 17.91 | 17.57 | 18.75 | 16.89 | 17.13 | 16.87 |
| C9JEA3 | Sialidase-4 | NEU4 | 18.93 | 17.98 | 18.62 | 16.90 | 17.49 | 17.80 |
| A0A3B3IUB9 | Baculoviral IAP repeat-containing protein 6 | BIRC6 | 18.46 | 18.42 | 19.23 | 17.06 | 17.84 | 17.92 |
| Q9C0G0 | Zinc finger protein 407 | ZNF407 | 17.97 | 17.24 | 17.59 | 16.92 | 16.48 | 16.13 |
| H7BYF2 | Nucleoporin p58/p45 | NUP58 | 19.61 | 19.77 | 19.73 | 18.76 | 18.56 | 18.54 |
| Q96I60 | PFKM protein | PFKM | 17.72 | 18.15 | 18.33 | 17.10 | 17.06 | 16.83 |
| P35556 | Fibrillin-2 [Cleaved into: Fibrillin-2 C-terminal peptide] | FBN2 | 21.07 | 21.65 | 20.37 | 19.89 | 20.05 | 19.94 |
| B4Z1E1 | MHC class I antigen | HLA-A | 20.71 | 20.45 | 20.82 | 19.48 | 19.60 | 19.70 |
| A0A1W2PRB5 | KAT8 regulatory NSL complex subunit 1 | KANSL1 | 21.42 | 21.43 | 21.24 | 20.36 | 19.68 | 20.85 |
| Q9NUD5 | Zinc finger CCHC domain-containing protein 3 | ZCCHC3 | 19.06 | 18.69 | 18.70 | 18.18 | 17.92 | 17.16 |
| A4QMS1 | Hypothetical gene supported by BC067869 | - | 20.96 | 20.74 | 20.82 | 19.33 | 19.94 | 20.06 |
| A0A024R3U8 | Uncharacterized protein | - | 20.20 | 20.74 | 19.66 | 19.05 | 19.05 | 19.31 |
| B4DPB7 | Elongator complex protein 3 | ELP3 | 19.27 | 20.41 | 19.39 | 18.75 | 18.51 | 18.61 |
| B3KT33 | cDNA FLJ37552 fis, clone BRCAN2028319, highly similar to Chromo domain-helicase-DNA-binding protein 1 | - | 21.38 | 21.71 | 20.74 | 20.15 | 19.92 | 20.57 |
| A0A4P2SQW8 | MHC class I antigen | HLA-A | 20.64 | 20.71 | 20.91 | 19.13 | 20.27 | 19.69 |
| Q96N03 | V-set and transmembrane domain-containing protein 2-like protein | VSTM2L | 18.17 | 17.92 | 17.73 | 16.31 | 17.28 | 17.05 |
| A0A024R430 | Uncharacterized protein | LOC92691 | 20.29 | 21.17 | 21.31 | 19.77 | 20.10 | 19.73 |
| Q8TCG6 | UORF | - | 17.24 | 17.12 | 17.08 | 16.39 | 15.56 | 16.32 |
| K7EK45 | Polypyrimidine tract-binding protein 1 | PTBP1 | 20.60 | 21.14 | 21.33 | 20.23 | 19.38 | 20.29 |
| H0YAM7 | Receptor of-activated protein C kinase 1 | RACK1 | 17.98 | 18.51 | 18.13 | 17.08 | 16.77 | 17.63 |
| S4R3P5 | Corrinoid adenosyltransferase | MMAB | 18.19 | 18.11 | 17.63 | 16.57 | 16.98 | 17.24 |
| A0A087X183 | La-related protein 4B | LARP4B | 21.49 | 21.57 | 21.36 | 19.88 | 20.67 | 20.74 |
| A0A6B9V3S8 | MHC class I antigen | HLA-B | 17.54 | 17.96 | 17.57 | 16.08 | 16.73 | 17.16 |
| M0QY96 | Heterogeneous nuclear ribonucleoprotein M | HNRNPM | 20.66 | 20.78 | 21.34 | 19.47 | 20.11 | 20.12 |
| Q96Q06 | Perilipin-4 | PLIN4 | 19.49 | 19.67 | 20.26 | 18.32 | 19.21 | 18.82 |
| Q5TZZ9 | Annexin | ANXA1 | 21.21 | 21.17 | 21.54 | 20.18 | 20.48 | 20.21 |
| P25391 | Laminin subunit alpha-1 | LAMA1 | 18.15 | 18.36 | 17.77 | 17.13 | 17.10 | 17.01 |
| Q9P227 | Rho GTPase-activating protein 23 | ARHGAP23 | 20.98 | 21.21 | 20.80 | 19.55 | 20.52 | 19.87 |
| B4DKE3 | cDNA FLJ60628, highly similar to Protein FAM5B | - | 21.85 | 21.26 | 21.30 | 20.45 | 20.72 | 20.21 |
| F8WCY1 | Transmembrane protein 44 | TMEM44 | 17.44 | 16.94 | 16.70 | 16.14 | 15.55 | 16.37 |
| S4R341 | Nucleolar and coiled-body phosphoprotein 1 | NOLC1 | 18.32 | 18.79 | 17.95 | 17.52 | 17.44 | 17.10 |
| A0A5C2G7R4 | IGL c397_light_IGKV3-11_IGKJ5 (Fragment) | - | 20.57 | 20.72 | 21.61 | 22.15 | 21.70 | 22.26 |
| A0A590UJA6 | t-SNARE domain-containing protein 1 (Fragment) | TSNARE1 | 17.00 | 16.55 | 16.43 | 17.68 | 17.88 | 17.66 |
| P29120 | Neuroendocrine convertase 1 | PCSK1 | 16.36 | 15.26 | 15.48 | 16.61 | 16.62 | 17.15 |
| A0A024R6A0 | Arginase (EC 3.5.3.1) | ARG2 | 20.69 | 20.72 | 20.94 | 21.83 | 21.95 | 21.85 |
| F5GXD9 | Integral membrane protein GPR137 (Fragment) | GPR137 | 16.13 | 16.07 | 16.72 | 17.32 | 17.45 | 17.59 |
| Q7Z4F4 | MSTP083 | - | 18.03 | 17.70 | 18.49 | 19.47 | 19.48 | 18.76 |
| B4DPR2 | cDNA FLJ50830, highly similar to Serum albumin | - | 18.08 | 18.95 | 18.72 | 20.46 | 19.30 | 19.87 |
| Q8N519 | LMNA protein | - | 18.01 | 16.02 | 17.28 | 18.96 | 18.52 | 18.81 |
| Q6ZWG5 | FLJ41131 protein | C19orf54 | 14.04 | 15.73 | 14.61 | 16.68 | 17.21 | 16.10 |
| Q2UY09 | Collagen alpha-1(XXVIII) chain | COL28A1 | 15.95 | 16.87 | 15.68 | 17.50 | 18.57 | 18.09 |
| B3KSM6 | cDNA FLJ36606 fis, clone TRACH2015654, highly similar to HEAT SHOCK 70 kDa PROTEIN 6 | - | 14.69 | 13.93 | 13.08 | 17.13 | 15.80 | 16.70 |
| A2RUE7 | Glutamate receptor | GRIN3A | 14.75 | 15.21 | 15.47 | 18.59 | 17.60 | 19.09 |
| Q9P2K3 | REST corepressor 3 | RCOR3 | 15.45 | 14.68 | 15.99 | 17.36 | 19.63 | 19.46 |
| A0A6Q8PFT7 | Ligand-dependent nuclear receptor corepressor-like protein (Fragment) | LCORL | 0.00 | 0.00 | 0.00 | 14.43 | 16.39 | 11.65 |
| Q8NA70 | Protein FAM47B | FAM47B | 0.00 | 0.00 | 0.00 | 14.81 | 14.74 | 15.70 |
| Q92618 | Zinc finger protein 516 | ZNF516 | 0.00 | 0.00 | 0.00 | 15.84 | 15.51 | 14.88 |
| B1ALG1 | Probable global transcription activator SNF2L2 | SMARCA2 | 0.00 | 0.00 | 0.00 | 15.14 | 15.89 | 15.84 |
| E7EQR6 | T-complex protein 1 subunit alpha | TCP1 | 0.00 | 0.00 | 0.00 | 13.13 | 16.97 | 16.84 |
| Q13383 | RNA binding motif (Fragment) | RBM | 0.00 | 0.00 | 0.00 | 15.05 | 16.52 | 16.52 |
| H7C1T4 | SRSF protein kinase 3 (Fragment) | SRPK3 | 0.00 | 0.00 | 0.00 | 14.41 | 16.88 | 17.16 |
| P11229 | Muscarinic acetylcholine receptor M1 | CHRM1 | 0.00 | 0.00 | 0.00 | 13.28 | 16.51 | 19.16 |
| A0A0C4DGH6 | UHRF1-binding protein 1-like | UHRF1BP1L | 0.00 | 0.00 | 0.00 | 16.20 | 16.94 | 16.31 |
| D3YRA6 | GDF5 (Fragment) | GDF5 | 0.00 | 0.00 | 0.00 | 16.43 | 16.51 | 16.53 |
| Q6PIE7 | MIA2 protein (Fragment) | MIA2 | 0.00 | 0.00 | 0.00 | 15.83 | 17.30 | 17.19 |
| A0A6E1W127 | Myosin-7B | MYH7B | 0.00 | 0.00 | 0.00 | 16.92 | 17.37 | 16.59 |
| Q6NSI4 | RPA-related protein RADX | RADX | 0.00 | 0.00 | 0.00 | 16.24 | 16.71 | 17.93 |
| A8MT33 | Synaptonemal complex central element protein 1-like | SYCE1L | 0.00 | 0.00 | 0.00 | 16.70 | 16.20 | 18.36 |
| A0A024R4W7 | Carnitine O-palmitoyltransferase (EC 2.3.1.21) | hCG_2044796 | 0.00 | 0.00 | 0.00 | 17.54 | 17.44 | 16.38 |
| M0QZ97 | Dipeptidyl peptidase 9 | DPP9 | 0.00 | 0.00 | 0.00 | 16.11 | 18.19 | 17.12 |
| A0A0E3D6M1 | Membrane-associated guanylate kinase, WW and PDZ domain-containing protein 2 | MAGI2 | 0.00 | 0.00 | 0.00 | 18.00 | 16.69 | 16.99 |
| A0A590UJD6 | Egl nine homolog 1 (Fragment) | EGLN1 | 0.00 | 0.00 | 0.00 | 16.12 | 17.84 | 17.73 |
| A0N0X8 | Hydroperoxy icosatetraenoate dehydratase (EC 1.14.14.1) (EC 4.2.1.152) | CYP1A1 | 0.00 | 0.00 | 0.00 | 17.22 | 17.71 | 17.18 |
| A0A024R0Z6 | Limb region 1 homolog (Mouse)-like, isoform CRA_a | LMBR1L | 0.00 | 0.00 | 0.00 | 17.17 | 17.34 | 18.08 |
| B4DUE0 | Tetratricopeptide repeat protein 38 | TTC38 | 0.00 | 0.00 | 0.00 | 18.57 | 20.12 | 19.38 |
| Q8IYQ7 | Threonine synthase-like 1 (TSH1) | THNSL1 | 0.00 | 0.00 | 0.00 | 18.60 | 19.81 | 20.60 |
| D2WEZ3 | Neurogenic locus notch homolog protein 2 (Neurogenic locus notch-like protein 2) | NOTCH2 | 0.00 | 0.00 | 0.00 | 18.18 | 20.36 | 20.83 |
| A0A1L4BJA8 | Polypeptide N-acetylgalactosaminyltransferase (EC 2.4.1.-) | GALNT13 | 0.00 | 0.00 | 0.00 | 20.18 | 19.90 | 20.36 |
| A0A0A0MRV7 | Protein-associated with UVRAG as autophagy enhancer | RUBCNL | 0.00 | 0.00 | 0.00 | 19.46 | 20.81 | 20.57 |
