# Supplementary material for: Quantitative proteomics analysis reveals possible anticancer mechanisms of 5’-deoxy-5’-methylthioadenosine in cholangiocarcinoma cells
Source: PLoS One. 2024 Jun 26;19(6):e0306060. doi: 10.1371/journal.pone.0306060 (PMC11206958; doi:10.1371/journal.pone.0306060)
Supplement: S1 Raw images — MS proteomics data. https://repository.jpostdb.org/entry/JPST002497; (PXID: PXD049119). (PDF) [file pone.0306060.s003.pdf]

Original blot images used for the generation of Figure 8C. All images were captured with an Alliance® Q9-ATOM Chemiluminescence Imager (Uvitec, Cambridge, UK).

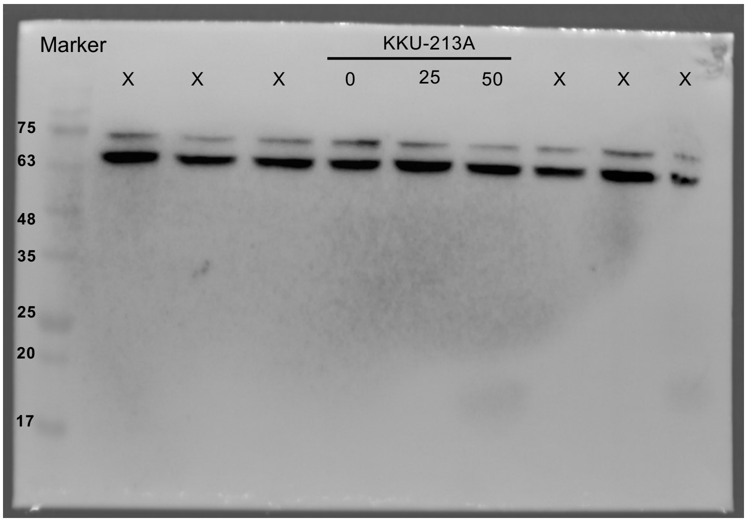

Protein from cell line KKU-213A treated with MTA at 0, 25, and 50 µg/ml, loaded at 30 µg/ml. Primary antibody: KLC1 (1:1,000 dilution, Abcam, Cambridge, UK).

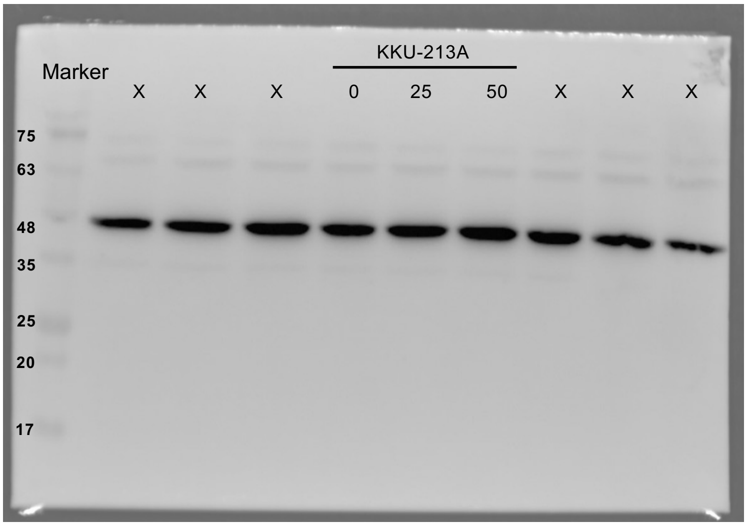

Protein from cell line KKU-213A treated with MTA at 0, 25, and 50 µg/ml, loaded at 30 µg/ml. Primary antibody: β-actin (1:10,000 dilution, Sigma-Aldrich, Darmstadt, Germany).

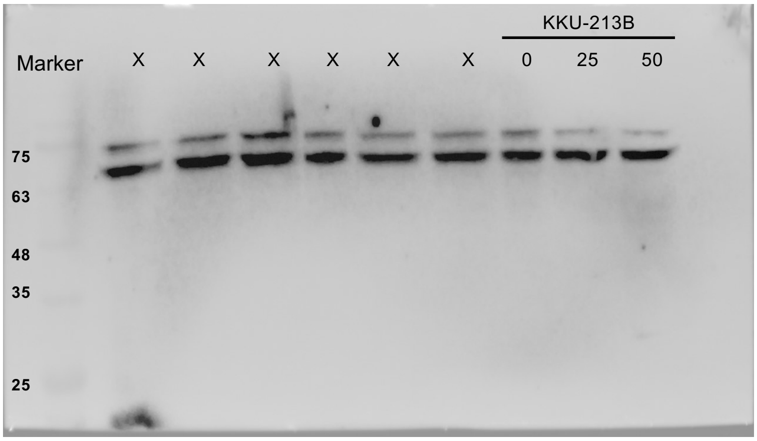

Protein from cell line KKU-213B treated with MTA at 0, 25, and 50 µg/ml, loaded at 30 µg/ml. Primary antibody: KLC1 (1:1,000 dilution, Abcam, Cambridge, UK).

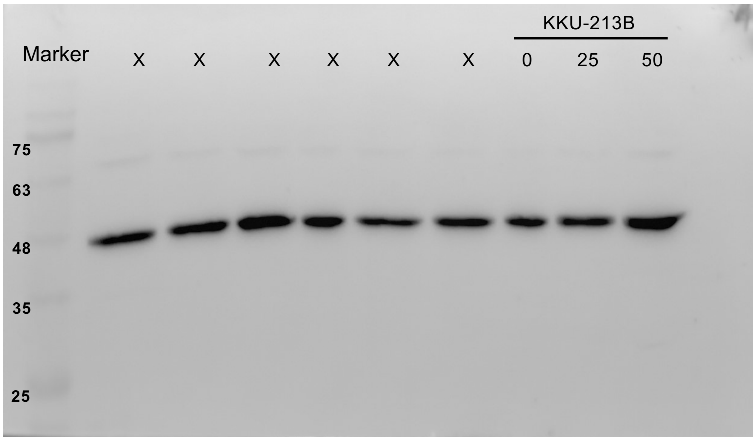

Protein from cell line KKU-213B treated with MTA at 0, 25, and 50 µg/ml, loaded at 30 µg/ml. Primary antibody: β-actin (1:10,000 dilution, Sigma-Aldrich, Darmstadt, Germany).
